# Analyzing the 2019 Chilean social outbreak: Modelling Latin American economies

**DOI:** 10.1371/journal.pone.0256037

**Published:** 2021-08-18

**Authors:** Sergio Curilef, Diego González, Carlos Calderón

**Affiliations:** 1 Departamento de Física, Universidad Católica del Norte, Antofagasta, Chile; 2 Banco Itaú-Corpbanca, Santiago, Chile; 3 Escuela de Psicología, Universidad Católica del Norte, Antofagasta, Chile; Institute for Economic Forecasting, Romanian Academy, ROMANIA

## Abstract

In this work, we propose a quantitative model for the 2019 Chilean protests. We utilize public data for the consumer price index, the gross domestic product, and the employee and per capita income distributions as inputs for a nonlinear diffusion-reaction equation, the solutions to which provide an in-depth analysis of the population dynamics. Specifically, the per capita income distribution stands out as a solution to the extended Fisher-Kolmogorov equation. According to our results, the concavity of employee income distribution is a decisive input parameter and, in contrast to the distributions typically observed for Chile and other countries in Latin America, should ideally be non-negative. Based on the results of our model, we advocate for the implementation of social policies designed to stimulate social mobility by broadening the distribution of higher salaries.

## Introduction

Partial differential equations and their solutions are used by specialists to account for certain physical and mathematical phenomena involved in modeling the behavior of several complex systems. For example, the reaction-diffusion equation is a serious candidate for analyzing the growth and spread of singular populations [1]. Other related nonlinear evolution equations are applied in fields such as ecology [2] and archaeology [3]. In particular, equations involving nonlinear diffusion terms were used in pioneering work in the field of astrobiology [4]. In addition, the extended Fisher–Kolmogorov equation [5] is a widely applicable tool that has provided valuable insights in statistical physics [6], nonlinear optics [7–9], quantitative biology [1], and finance [10, 11], among other areas [12–15]. The suitability of evolution equations that utilize porous diffusion as the nonlinear diffusion term has long been debated as they often permit analytical solutions exhibiting a maximum q-entropy form [16]. Under proper simple constraints [6, 17], such solutions play a prominent role in novel applications of evolution equations involving nonlinear, power-law diffusion.

### Social context

Recently, a multifaceted economic problem has emerged in Latin American countries. Claims of inequality, inequity, inadequate healthcare, and a lack of education resources are just some of the subjects stimulating public protests. Contrary to common belief, evaluating the life quality of citizens in a particular society is no longer related solely to wealth production [18, 19]. Instead of economic growth, citizens’ life quality is related to the level of perceived income inequality [20]. Despite significant economic development in recent decades leading to a substantial decrease in poverty levels, economic success has not necessarily translated to improvements in social indicators such as life expectancy, educational performance, and high-quality healthcare. For certain demographics, the key factor for measuring well being is not income but their perceptions of subjective factors such as inequality. Considering the relative deprivation theory as a frame of reference, several studies [21–23] support the argument that self-perception of social status is a key psychological mechanism for studying the relationship between inequality and the subjective perception of well-being. A recent study conducted on a large sample of the European population reported that income inequality has an indirect effect on the perception of wellbeing, with subjective social status used as a mediating variable [24]. The consensus is that greater income inequalities correspond to a decrease in subjective social status, which negatively impacts the perception of wellbeing. Additional studies have evaluated the respective influences of other mediating variables attributed as root causes of social conflicts, such as citizen trust, state anxiety, perception of lower social status, and a decrease in subjective wellbeing [25].

This work illustrates how the application of a typical physical equation enables multifactor problems to be analyzed effectively. Although motivated by studying the causes and effects of the social unrest in Chile, the proposed analytical approach is valid for any country, and particularly applicable to countries with similar income distributions, such as those in Latin America. Chile is an ideal case study because it best represents the economic indicators in the region, and we tested the model using its economic variables. Nevertheless, the model is sufficiently general to be applied to any country where the public data distributions required for the model input are known. As such, the main objective of this model is to provide an accurate assessment of the influence of population dissatisfaction. Although most people possess a better quality of life compared with the previous generation, many people feel themselves to be victims of an inequal society. Specifically, they believe that many vital products and services are unattainable for people belonging to their demographic. In Chile, over 50% of the population earns less than the mean income. This, combined with other aforementioned societal features, has caused general dissatisfaction among the population, resulting in sustained periods of civil unrest. This raises the following question: How can inequality be decreased?

### Economic elements

**Fig 1:**
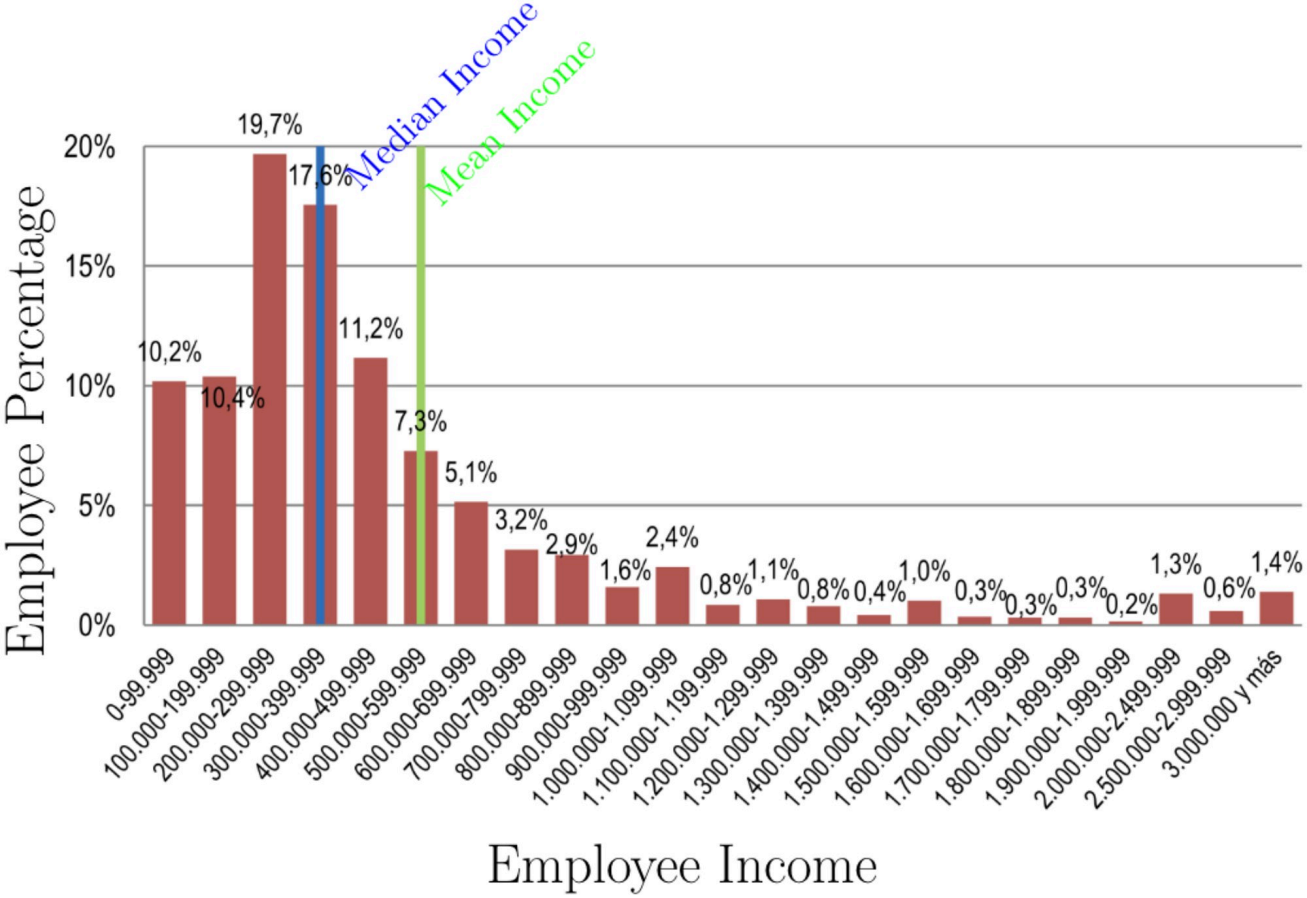
Employee percentage as a function of employee income. The mean income is between 500000 and 599000 CLP (Chilean pesos), while the median income is between 300000 and 399000 CLP; therefore, over 50% of the population lives on significantly less than the mean.

We used Chile as a case study to test our proposed analysis method as the indicators contributing to its macroeconomic state have been studied extensively, has and comprise political, psychological, economic, and ideological elements. In our model, more than any ideological or subjective factors, we consider the increase of the gross domestic product (GDP), which competes with the consumer price index (CPI) in an extended Fisher-Kolmogorov equation [26, 27]. Additionally, the increase in income and its distribution [28] (see Fig 1) are used to identify the dynamics of the demographic that live on a certain amount of money. The distributions and parameters used in the study are public data obtained from the official data base [28].

## Method and variables

The nonlinear diffusion equation is applied to complex systems designed to solve problems related to population growth, *W* [1, 6, 29–31]. Therefore, we consider a natural extension of this equation to incorporate density-dependent diffusion, *D*. For a simple one-dimensional scalar case, this extended equation is expressed as [1]

$$\begin{array}{c} \frac{\partial }{\partial t^{\prime }}W=\frac{\partial }{\partial x^{\prime }}(D\frac{\partial }{\partial x^{\prime }}W)+G, \end{array}$$ (1)

where *G* = *rW* (1 − *f*(*x*′, *t*′)*W*^*n*−1^) is a function that has two zeros, namely at *W* = 0 and *f*^1/(1 − *n*)^. In addition, *D* = *W*^*m*^/(*m* + 1) represents a nonlinear diffusion-reaction equation, which is usually used to model insect dispersal [1].

In addition, changing the variables $x^{\prime }=\sqrt{\frac{r}{\kappa }}x$, *t*′ = *rt*, and *f*(*x*, *t*) = *μ*(*t*)*c*(*x*)/*r*, while *m* + 1 = 2 − *q* and *n* = *q* leads to the extended Fisher-Kolmogorov equation [5, 17]:

$$\begin{array}{c} \frac{\partial }{\partial t}W=\frac{\kappa }{2-q}\frac{\partial^{2}}{\partial x^{2}}W^{2-q}+rW-\mu \left(t\right)c\left(x\right)W^{q}, \end{array}$$ (2)

where the parameters *r* and *κ* are positive and the functions *μ*(*t*) and *c*(*x*) are continuous and non-negative. Specifically, the reproduction rate is time-dependent, i.e., *r* = *r*(*t*), and is proportional to the existing population and available resources, while the competition *μ*(*t*) represents the available facilities, and the function *c*(*x*) accounts for the spatial dependence, which is related to the nonhomogeneous distribution of the local and limiting resources required in response to the population growth.

This scenario provides several possible models in which this nonlinear power-law diffusion term can explain the interaction between particles. For instance, the spatial density of a particle system engaged in overdamped motion under the effect of interactions described by forces that evolve according to the nonlinear diffusion equations [32].

Alternatively, *q* → 2 corresponds to a superdiffusive regime used to model multiple interactions between particles [32] in a complex system with the aim of determining the population dynamics that surprisingly appear in nonlinear optics problems [9]. Consequently, this choice recovers the original logistic population growth term representing the birth/death ratio in ecological studies. In this case, the equation is expressed as

$$\begin{array}{c} \frac{\partial }{\partial t}W=\kappa \frac{\partial^{2}}{\partial x^{2}}\log W+rW-\mu c\left(x\right)W^{2}, \end{array}$$ (3)

where *t* (years) and *x* (income) are independent variables. The function *c*(*x*) represents the employee income distribution and *W*(*x*, *t*) corresponds to the per capita income distribution, which evolves according to Eq (3); both of these distributions are normalized within an income range. Furthermore, the rate (*r* = *r*(*t*)) is related to the variation of the GDP, which is a monetary measure of the market value of all goods and services produced in a specific time period. On the other hand, the competition (*μ* = *μ*(*t*)) depends on the variation of the employee incomes, commonly adjusted by the CPI every year. By the way, the CPI measures the average change over time in the prices paid by urban consumers for a market basket of consumer goods and services. Both the GDP and CPI tend to be updated annually.

## Results

### Numerical approximation

Fig 1 shows employee percentage as a function of employee income according to the INE Chilean Report 2010-2015 [28]. This distribution is represented by a Lorentzian function given by

$$\begin{array}{c} c\left(x\right)=\frac{0.197}{1+0.222\times {\left(x-2.50\right)}^{2}}, \end{array}$$ (4)

where *x* = Income × 10^−5^ *CLP* with *x* ∈ [0, 50], with the employee percentage divided by 100 to normalize *c*(*x*) to 1, as $\int_{0}^{50}\;c\left(x\right)\;\text{d}x=1$. Moreover, the mode (at the 19.7% population interval), median, and mean incomes corresponds to *x* ∈ [2, 3), *x* ∈ [3, 4), and *x* ∈ [5, 6], respectively.

In addition, we define a distribution for the income per capita to represent deciles used to classify socioeconomic sectors according to the total money contributed by the members of a household, divided by the number of household members: decile 1 represents the population with the most vulnerable socioeconomic status, while decile 10 represents the population with the highest incomes. This distribution corresponds to a Lorentzian, i.e.,

$$\begin{array}{c} W\left(x,0\right)=\frac{0.359}{1+0.636\times {\left(x-1\right)}^{2}}, \end{array}$$ (5)

and is normalized in the same interval of *x*, $\int_{0}^{50}W\left(x,0\right)\;\text{d}x=1$. The evolution of *W*(*x*, *t*) reveals valuable information regarding the population’s perceptions.

When integrating the extended Fisher-Kolmogorov Eq (3), we consider the function *c*(*x*) as defined in Eq (4), with *r* = 0.04 and *μ* = 0.03. We focus on the stationary state, that is, when *t* → ∞; this is expressed as ∂*W*/∂*t* = 0, with the boundary conditions

$$\begin{array}{c} W\left(x\right)=\{\begin{array}{ccc} W_{0} & \text{if} & x=0,\\ 0 & \text{if} & x\to \infty , \end{array} \end{array}$$ (6)

In addition, *W*_0_ meets the normalization condition, i.e., ∫*W*(*x*)d*x* = 1, enabling a comparison with *W*(*x*, 0). In this approximation, we apply a type of global regulation mechanism, which conserves the total population [7], to satisfy the normalization condition and emphasize certain properties that characterize the distribution.

**Fig 2:**
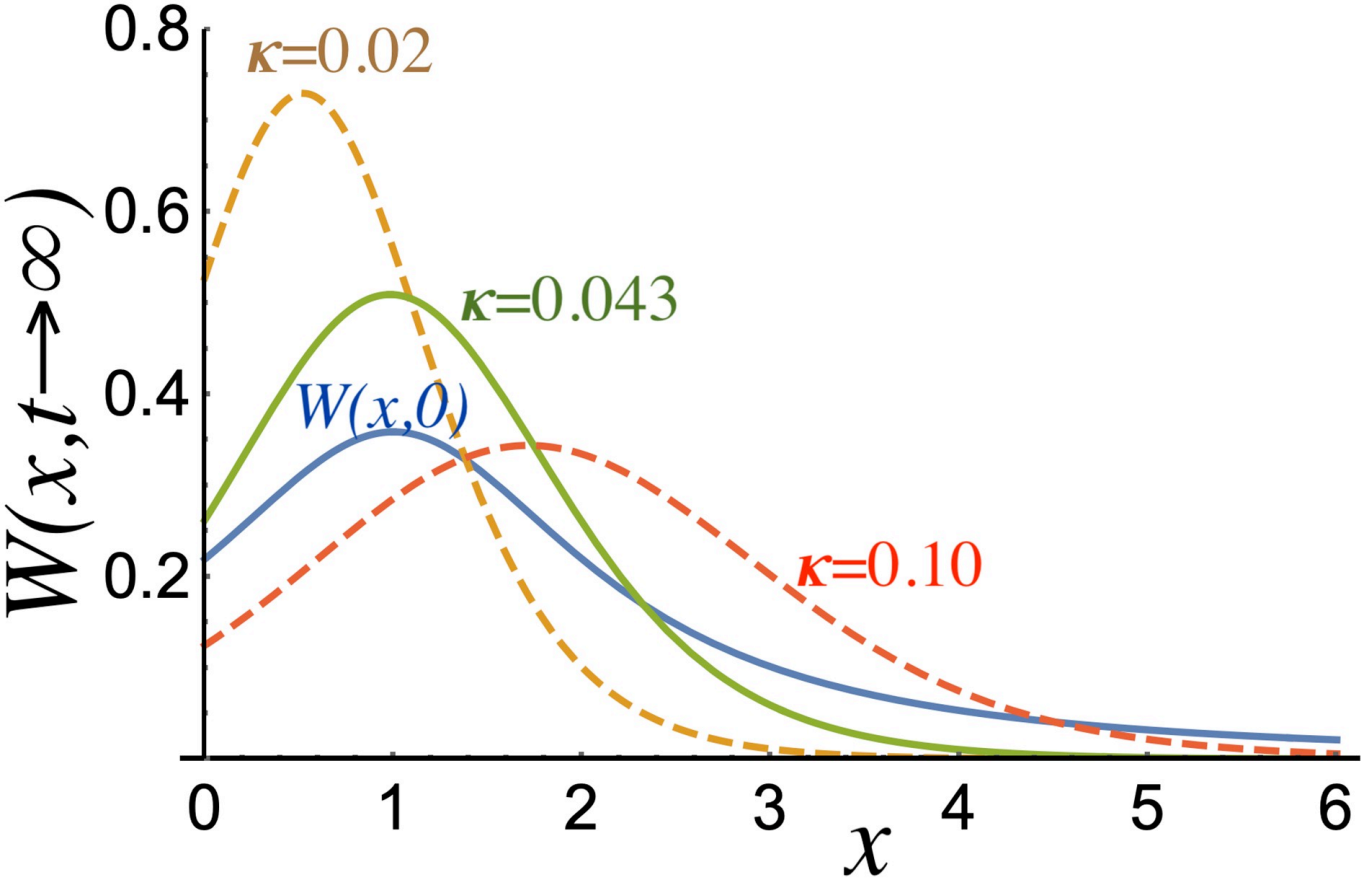
*W*(*x*, 0) as a function of *x* in comparison with normalized stationary solutions of Eq (3) for *κ* = 0.02, 0.043, and 0.1.

First, we assume that the constant *κ* has the same order of magnitude as *r* and *μ*. In terms of our results, if *κ* < 0.043, the mode of the function *W* satisfies *x* < 1, which means the distribution mode (i.e., the value that appears most often in the data set) decreases from its original value. Fig 2 shows three different stationary solutions together with the function *W*(*x*, 0). The curves correspond to specific cases in which the mode evolves at the same position (*x*) for *κ* = 0.043; relative to the initial mode, it decreases for *κ* = 0.02 and increases for *κ* = 0.1. According to our interpretation, a value of *κ* < 0.043 indicates that people have to live with increasingly less income, symbolizing an increase in poverty that becomes dangerous when people lose access to social security and see no prospect of their situation improving. This scenario appears to have manifested in Chilean society.

### Analytical proposal

Now, in accordance with the Plastino and Plastino-like formulations [17], we can build analytical solutions to the extended Fisher-Kolmogorov Eq (2) by adopting the following ansatz: $W\left(x,t\right)=a\left(t\right){\left(1-\left(1-q\right)\frac{{\left(x-y\left(t\right)\right)}^{2}}{4\sigma {\left(t\right)}^{2}}\right)}^{\frac{1}{1-q}},$ which maximizes the power-law q-entropy [16] for properly defined constraints [6, 17], where *σ*(*t*) represents the distribution width, *a*(*t*) is the distribution amplitude, and *y*(*t*) is the mode position. For simplicity, we use an alternative ansatz to solve the equation for *q* = 2 [5], namely

$$\begin{array}{c} W\left(x,t\right)={\left(A\left(t\right)+\frac{{\left(x-y\left(t\right)\right)}^{2}}{4S(t)}\right)}^{-1}, \end{array}$$ (7)

where *a*(*t*) = *A*(*t*)^−1^ is the amplitude function and *σ*^2^(*t*) = *A*(*t*)*S*(*t*) is the width of the distribution. Next, we replace the income distribution function with the empirical version stated in Eq (4), which leads to

$$\begin{array}{c} c\left(x\right)=h_{1}+h_{2}{\left(x-x_{p}\right)}^{2}, \end{array}$$ (8)

where *x*_*p*_ is the mode of the distribution and *h*_2_ defines the concavity of the parabola (8). Now, by substituting the simplified ansatz (7) and corresponding function (8) into Eq (3), we obtain a set of three coupled equations:

$$\begin{array}{ccc} S^{\prime }\left(t\right) & = & \frac{1}{2}\kappa +rS\left(t\right)-4h_{2}\mu S{\left(t\right)}^{2}, \end{array}$$ (9)

$$\begin{array}{ccc} y^{\prime }\left(t\right) & = & -4\mu h_{2}\left(y\left(t\right)-x_{p}\right)S\left(t\right), \end{array}$$ (10)

$$\begin{array}{ccc} A^{\prime }\left(t\right) & = & \kappa \frac{A(t)}{2S(t)}-rA\left(t\right)+\mu h_{1}+\mu h_{2}{\left(y\left(t\right)-x_{p}\right)}^{2}, \end{array}$$ (11)

where the Eq (9) is known as the *Riccatti equation* and the other equations represent special cases of the Bernoulli equation. Thus, the set of equations is solved exactly using suitable initial conditions. Taking the initial condition *S*_0_ = *S*(0), we have

$$\begin{array}{c} \begin{array}{cc} S(t)= & \frac{\sqrt{8h_{2}\kappa \mu +r^{2}}\tanh(\frac{1}{2}\left(t-t_{1}\right)\sqrt{8h_{2}\kappa \mu +r^{2}})+r}{8h_{2}\mu }\\ t_{1}= & \frac{2\;}{\sqrt{8h_{2}\kappa \mu +r^{2}}}\text{arctanh}(\frac{r-8h_{2}\mu S_{0}}{\sqrt{8h_{2}\kappa \mu +r^{2}}}). \end{array} \end{array}$$

**Fig 3:**
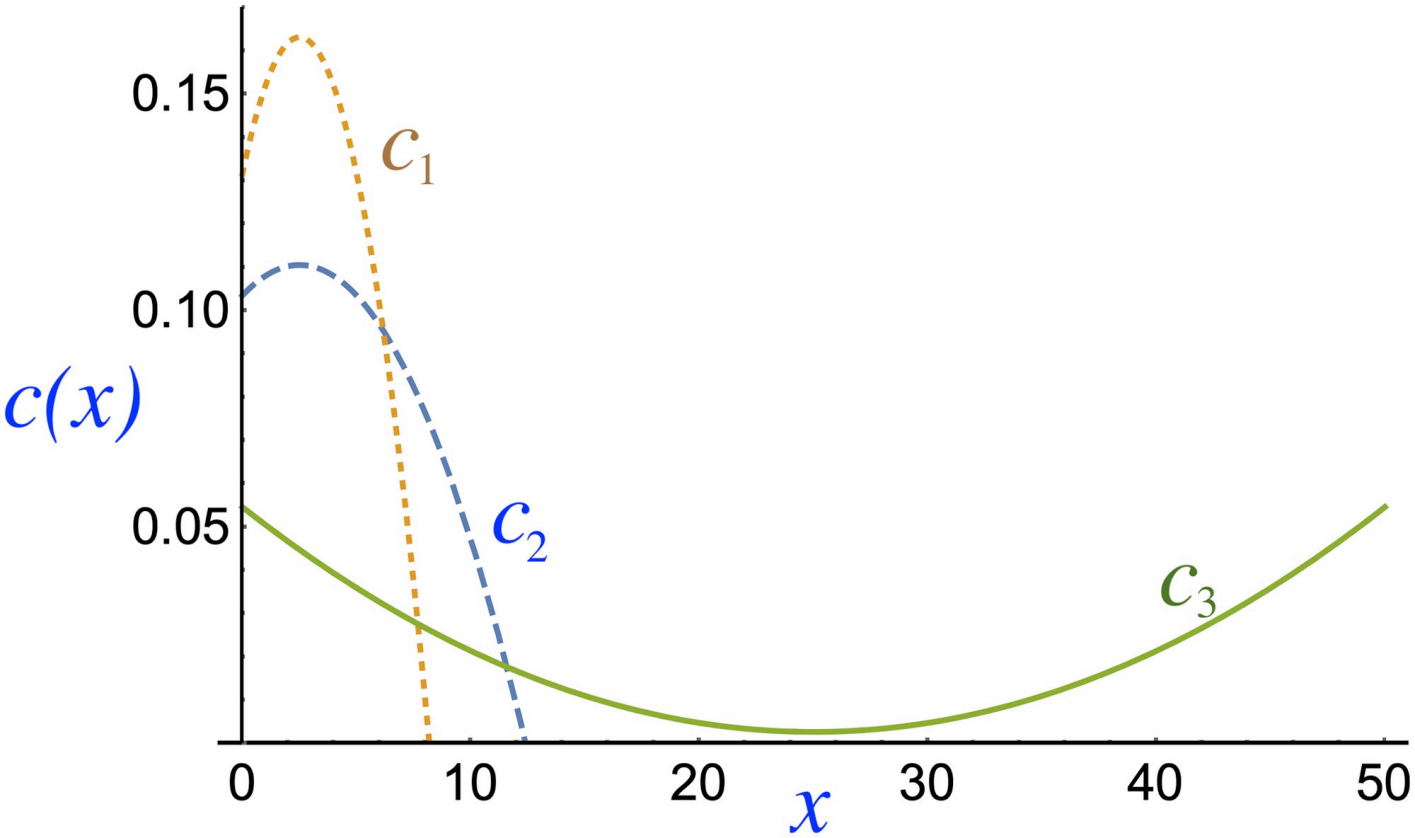
Three analytical functions *c*_*i*_(*x*) (where *i* = 1,2,3) are proposed as alternatives to the exact function *c*(*x*). Dotted/dashed and solid lines represent concave and convex functions, respectively.

As Eq (9) is solved, the solution of the equation set (9–11) can be found. For instance, certain quadratic profiles (8) can now be tested in place of the exact function *c*(*x*), such as

$$\begin{array}{ccc} c_{1}\left(x\right) & = & 0.163-5.04\times 10^{-3}{\left(x-2.5\right)}^{2}\\ c_{2}\left(x\right) & = & 0.111-1.13\times 10^{-3}{\left(x-2.5\right)}^{2}\\ c_{3}\left(x\right) & = & \left(2.69+0.0831{\left(x-25\right)}^{2}\right)\times 10^{-3} \end{array}$$ (12)

where *x* = Income × 10^−5^. These quadratic profiles are shown in Fig 3.

At this stage, we propose two downward concave parabolas (*h*_2_ < 0) in *c*_1,2_ and one upward parabola (*h*_2_ ≥ 0) in *c*_3_. The median of [*c*_1_(*x*)] is 3.23 and the mean of [*c*_2_(*x*)] is 4.87, which equal the median and mean values of *c*(*x*), which imposes ∫*c*_*i*_(*x*)d*x* = 1, with *h*_2_ = −5.04 × 10^−3^ and −1.13 × 10^−3^, respectively. Additionally, in contrast to *c*_1_ and *c*_2_, the function *c*_3_(*x*) represents a nontrivial income distribution that features a concavity, which represents a solution to the inequality problem because the distribution is a concave upward parabola. For example, if this distribution is symmetric, the population may perceive that the possibility of obtaining a low or a high salary is equally likely. The perception of the population is a subjective component of the problem, but it may be decisive when measuring the relationship between perceived dissatisfaction and social status.

## Discussion

**Fig 4:**
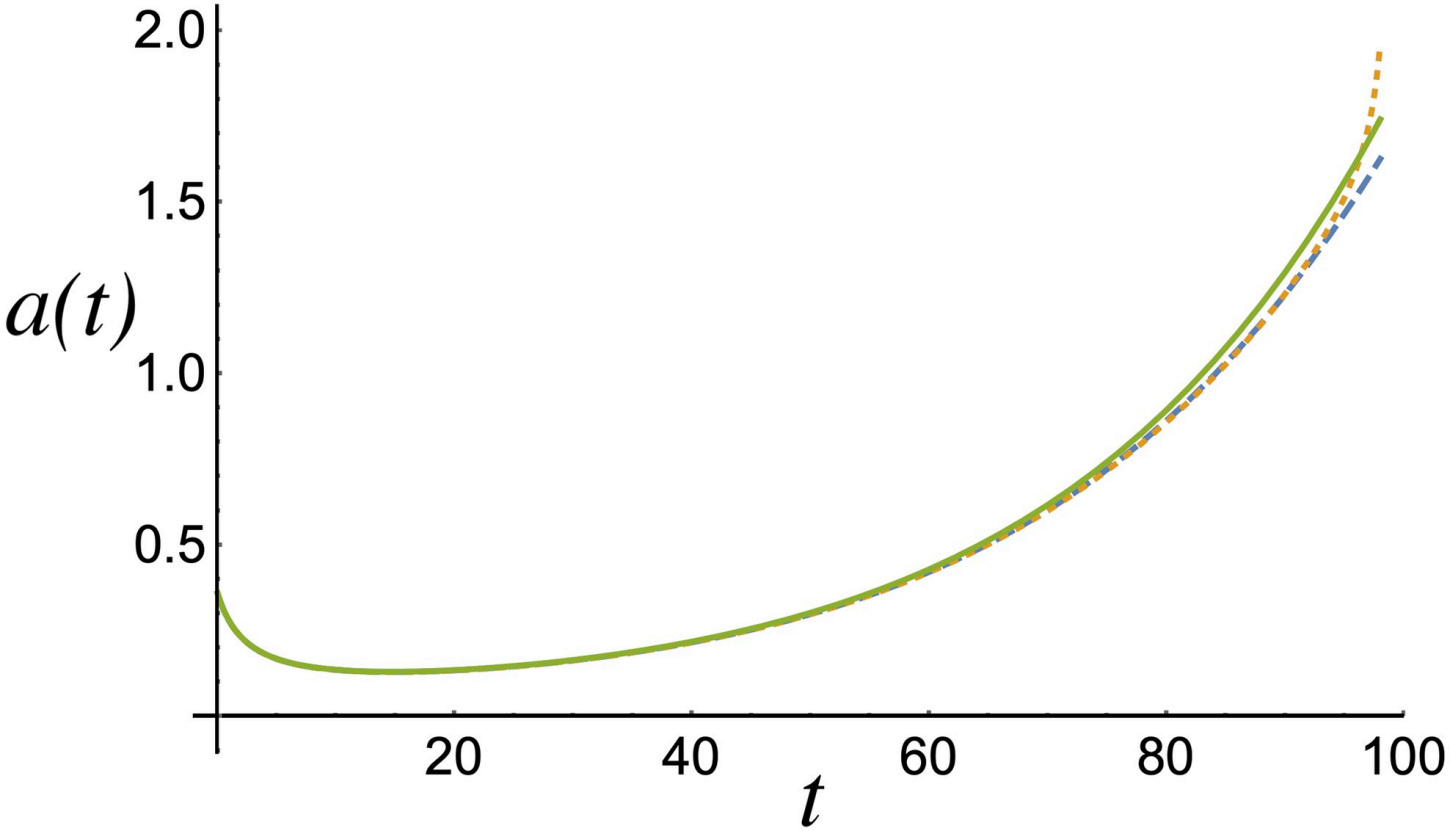
Amplitude of *W*(*x*, *t*), i.e., *a*(*t*) = 1/*A*(*t*) as a function of time. In each case, the amplitude increases as time progresses. The dotted, dashed, and solid lines represent the amplitudes associated with *c*_1_(*x*), *c*_2_(*x*), and *c*_3_(*x*), respectively, for *h*_2_ > 0.

**Fig 5:**
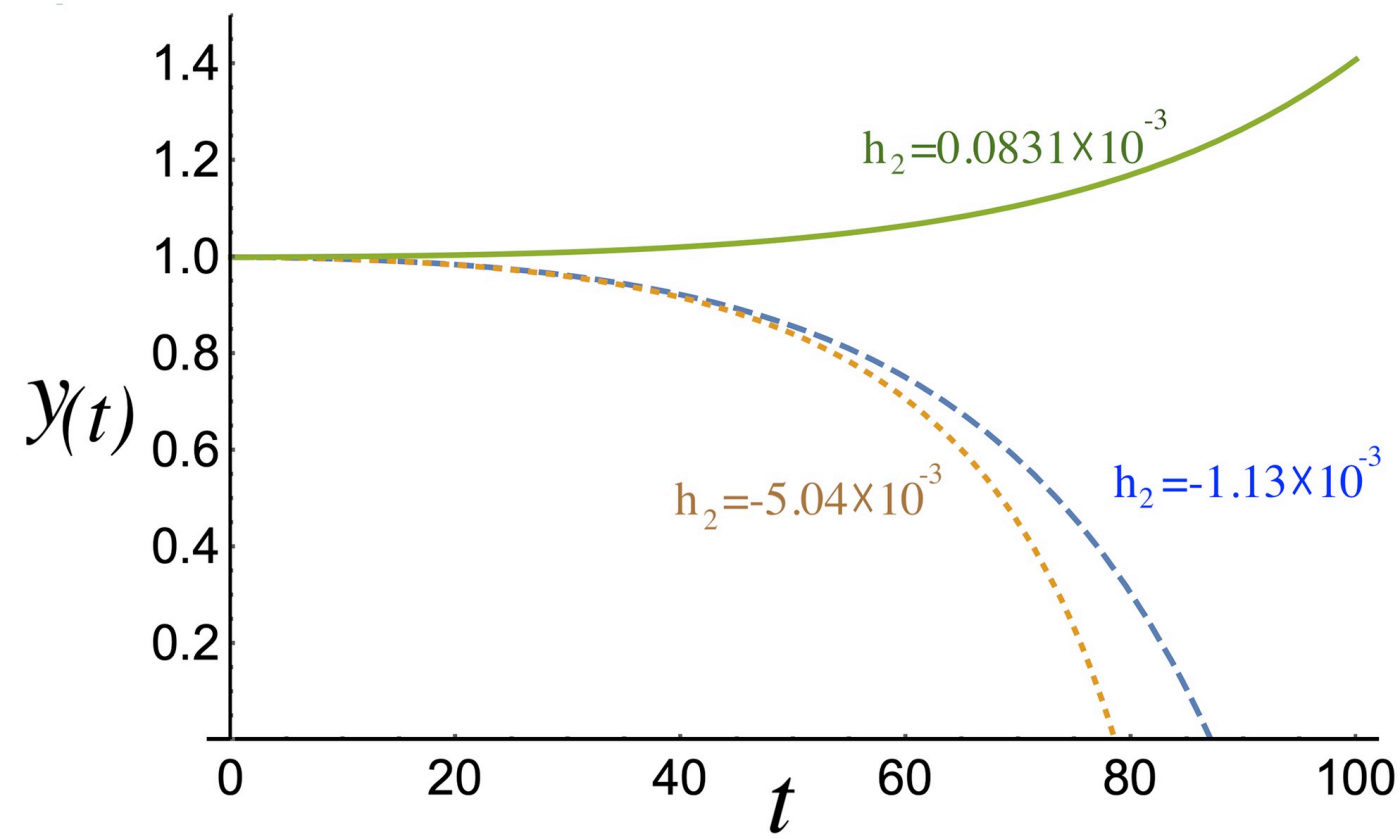
Comparison of the evolution of *y*(*t*) for different employee income concavities. The mode of *W*(*x*, *t*) is *y*(*t*) and, if *h*_2_ < 0, over time it tends to zero. Conversely, if *h*_2_ > 0, *y*(*t*) diverges from zero. The dotted, dashed, and solid lines represent the evolution of *y*(*t*) corresponding to *c*_1_(*x*), *c*_2_(*x*), and *c*_3_(*x*), respectively, for *h*_2_ > 0.

Fig 4 shows the amplitude *a*(*t*) = 1/*A*(*t*) as a function of time, revealing the proportion of the population that lives within the distribution peak. The evolution of the amplitude, which depends on the concavity of the growth of the total population, shows that it remains fairly consistent across the measured intervals. Then, the most relevant property is clear from the evolution of *y*(*t*) (Fig 5), which shows how the population peak shifts from its initial position to zero in the case where the income distribution has a downward concavity (*c*_1,2_(*x*)) resembling the empirical *c*(*x*). This feature is critical because it indicates that the incomes of a large number of people are decreasing. Initially, this process evolves slowly; however, it noticeably accelerates from year 50, where the perception is worsened, and totally collapses between years 80 and 90 based on the data used here. Different behavior is obtained for *h*_2_ ≥ 0, where the proposed function *c*_3_(*x*) tends to be uniform or displays a minimum but never displays a maximum, as in the previous case. This means that the evolution of *y*(*t*) shows an increase in the per capita income, *W*(*x*, *t*), resulting in rising incomes and an increased perception of social mobility. Therefore, the dynamics of the mode of the distribution *W*(*x*, *t*) are decisive in understanding social behavior when a population challenges evident inequality.

Besides, by the Eq (9), the stationary state, i.e., the *t* → ∞ limit, is well-defined in our analytical proposal only if *r*^2^ + 8*h*_2_ *κμ* ≥ 0. If *h*_2_ > 0, the conditions is always satisfied because all parameters are positive; however, if *h*_2_ < 0, the condition drops to *κ* < *r*^2^/8|*h*_2_|*μ*. Let us take the special case where no diffusion process is considered in the economic phenomena, this means that *κ* → 0, which leads the Eq (3) to the stationary solution given by *W*(*x*)→*r*/*μc*(*x*). Hence, the diffusion parameter *κ* ≠ 0 induces an acceleration in the dynamics of economic phenomena. As seen before, when *h*_2_ < 0 in the analytical *c*(*x*), the stationary solution is not relevant because the variable *y*(*t*) falls to zero, as illustrated in Fig 5. Solutions for *y*(*t*) < 0 do not represent any economic situation. Nevertheless, if *h*_2_ > 0, all solutions are relevant, and the stationary state is reached and well described by the present analytical proposal.

According to our model, the notion of gap shortening consists of defining a narrower income interval. In this scenario, salaries need not be drastically different. Equity and ethics are concepts that we can draw on here as a strategy to decrease inequality, where employee income is, at least, uniformly distributed between a minimum and a maximum value regulated by proper public policies. As demonstrated by our modeling, the shape of the distribution *c*(*x*) strongly affects the dynamics.

Latin America, and particularly Chile, has significant levels of social insecurity. Even when macroeconomic analyses show indicators of success, much of the population remains dissatisfied. Economic growth, together with an increase in per capita income (which neglects the adequate distribution of resources), can lead to a perceived increase in inequality.

Our results can inform the design of new public policies:

1. The interval between the minimum and maximum incomes must be redefined to introduce concepts such as ethical income, where the rate between the maximum and minimum values constitutes a relevant parameter.
2. Employee income dispersion may be reduced compared to current levels. For instance, the maximum income should not exceed ten times the minimum. This measure can decrease inequality.
3. Employee income distribution needs to be uniform in the interval between the minimum and maximum incomes or be defined according to other curves with upward concavity. However, there should never be a downward concavity.
4. A single peak in the function *c*(*x*) should be defined as representing absolute equality of income, for example, *h*_2_ ≪ 0, which represents a short evolution time for the function *y*(*t*) according to Fig 5. This indicates a rapidly collapsing economy and has an adverse effect on the per capita income.

In addition, previous studies have shown a close correlation between increasing debt and increasing inequality [20, 33, 34]; as a consequence, people outside the wealthiest category find it increasingly difficult to maintain their income or fulfill their aspirations. Subsequent studies should include additional indicators to provide more accurate explanations for the social unrest [33, 34].

Finally, we emphasize that, while linearity has previously provided sufficiently accurate explanations for phenomena in physics and other disciplines, many phenomena in nature demonstrate nonlinear behaviors. Therefore, we propose an exact analytical solution to the Fisher-Kolmogorov equation for tackling problems contingent on multiple parameter combinations using a power-law ansatz. As the standard Fisher-Kolmogorov equation is a continuity equation, the total population size is not conserved. Consequently, this model incorporates a global regulation mechanism that fixes the total population size in the numerical example, thereby enabling the initial distribution to be compared to the final one. Thus, the extent to which certain relevant properties deviate from the initial condition can be determined; for instance, the distribution maximum, which indicates the increase in the proportion of the population living on the mode income. In all cases, the peak seems to be higher. Furthermore, we emphasize that coupled and ordinary differential equations can be used to obtain dynamic solutions, thereby overcoming the limitations of linearity. The effectiveness of this method has been demonstrated for many natural phenomena that exhibit nonlinear behavior. Therefore, specialists must focus on developing families of nonlinear diffusion equations that can be applied to the modeling of nonlinear phenomena.
